# Luminescent Metal Complexes as Emerging Tools for Lipid Imaging

**DOI:** 10.1007/s41061-022-00400-x

**Published:** 2022-08-17

**Authors:** Bradley J. Schwehr, David Hartnell, Massimiliano Massi, Mark J. Hackett

**Affiliations:** 1 School of Molecular and Life Sciences, Curtin University, Perth, WA 6845 Australia; 2 Curtin Health Innovation Research Institute, Curtin University, Perth, WA 6845 Australia

**Keywords:** Metal complexes, Lipid droplets, Phospholipids, Fluorescence, Microscopy, Cell imaging

## Abstract

Fluorescence microscopy is a key tool in the biological sciences, which finds use as a routine laboratory technique (e.g., epifluorescence microscope) or more advanced confocal, two-photon, and super-resolution applications. Through continued developments in microscopy, and other analytical methods, the importance of lipids as constituents of subcellular organelles, signalling or regulating molecules continues to emerge. The increasing recognition of the importance of lipids to fundamental cell biology (in health and disease) has prompted the development of protocols and techniques to image the distribution of lipids in cells and tissues. A diverse suite of spectroscopic and microscopy tools are continuously being developed and explored to add to the “toolbox” to study lipid biology. A relatively recent breakthrough in this field has been the development and subsequent application of metal-based luminescent complexes for imaging lipids in biological systems. These metal-based compounds appear to offer advantages with respect to their tunability of the photophysical properties, in addition to capabilities centred around selectively targeting specific lipid structures or classes of lipids. The presence of the metal centre also opens the path to alternative imaging modalities that might not be applicable to traditional organic fluorophores. This review examines the current progress and developments in metal-based luminescent complexes to study lipids, in addition to exploring potential new avenues and challenges for the field to take.

## Introduction

Lipids are ubiquitous across all cell systems, serving a wide range of biological functions; for example, they modulate cell and organelle membrane structure and fluidity, are a vital metabolic energy source, and hold important inflammatory signalling roles [1, 2]. Yet, despite the established importance of lipids, many specific pathways of lipid synthesis, storage and metabolism remain only partly understood. Apart from key roles for healthy tissue and cell function, lipids may be central to disease pathways, and indeed lipid biology is implicated in inflammatory disorders [3–5], neurodegenerative diseases [1, 6, 7] and conditions that result from lysosomal storage disorders [8–10]. With increasing recognition of the role that lipids hold in the fundamental function and health of cells and tissues, it is critical to develop robust and accessible imaging tools to study lipid biology at the cellular and subcellular level.

**Fig. 1:**
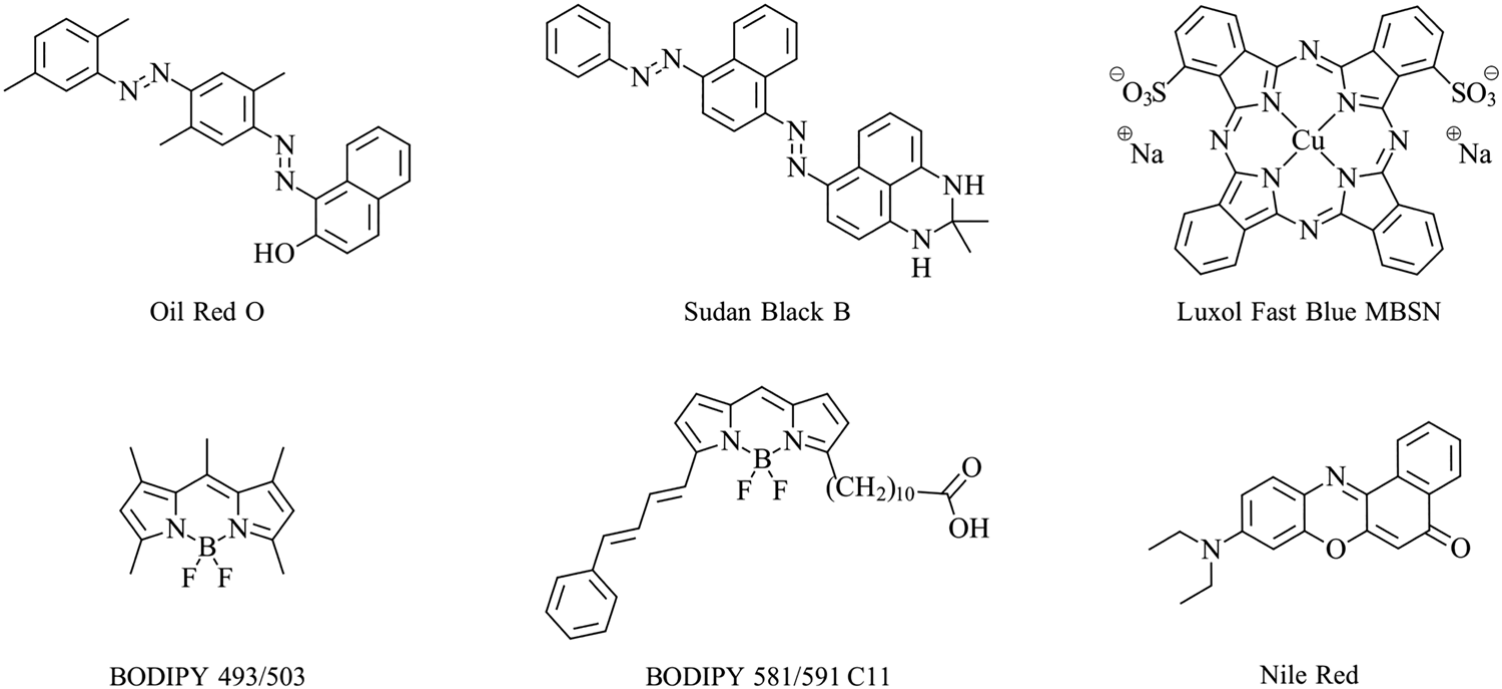
Chemical structures of traditional histochemical dye examples, commercially available BODIPY dyes, and Nile Red

Traditional histochemical methods typically incorporate lipophilic dyes (stains) such as Oil Red O [11, 12], Sudan Black B [13, 14] and Luxol Fast Blue [15] (Fig. 1), and have long been used to study lipid distribution in cells and tissues. Such methods have provided and continue to provide substantial and valuable insight into lipid biology. Unfortunately, the majority of these stains are not compatible for imaging live cells and are only applicable to chemically fixed cells and tissues. Further, histochemical methods offer relatively limited options for visualising lipid co-localisation with other biochemical-, organelle- or cell-specific markers. Detection limits associated with light microscopy and histochemical stains are also typically poorer than fluorescence microscopy methods, which can largely be attributed to the compatibility of the latter with the use of lasers. Lastly, while histochemical methods are well suited to detect neutral lipids, they are not well suited to detect polar lipids or to differentiate between lipid classes.

More recently, fluorescent probes have enabled scientists to greatly expand the fundamental knowledge of lipid biology. Boron-dipyrromethene-based molecular probes, more widely known as BODIPY (Fig. 1), have paved the way for countless explorations of lipid biochemistry in cells. BODIPY-based probes are relatively easy to solubilise in cell culture media and are generally compatible with live cell imaging. They have been successfully developed to provide key insight into lipid biosynthesis [16] and the connection of lipid droplets to the endoplasmic reticulum (ER) [17], lipid droplet dynamics at early stages of bacterial infection [18], lipid droplet function in skeletal muscle [19, 20], analysing endocytic sterol trafficking [21], and visualisation and quantification of neutral lipid content in various microalgae [22, 23], mammalian cells [24, 25] and other organisms [26, 27]. Commercially available BODIPY 581/591 C11 (Fig. 1) is used as a lipid peroxidation sensor in cells and membranes [28, 29]. More recently, other BODIPY analogues have been shown to be compatible with lipid imaging in ex vivo tissue sections, opening new opportunities to study lipid biology in animal models [30]. Another cause for recent excitement in the field of lipid-tissue imaging was the re-examination of Nile Red (Fig. 1), finding use as a fluorescent stain of myelin lipids during multiple sclerosis [31].

While BODIPY-based probes, along with the previously mentioned stains, are currently of widespread application to study lipids in cells, these compounds are still associated with some drawbacks that are intrinsic to their organic chemical nature [32]. The photophysics of organic compounds is usually characterised by typically small Stokes shifts, meaning that their absorption and emission profiles are overlapping to some extent [33]. This overlap can cause concentration quenching by reabsorption of emitted photons, especially when the local concentration of the probe increases due to preferential localisation in specific regions of the cell. Another typical issue often associated with organic fluorophores is photo-bleaching [34]. The amount of energy added to the fluorophore during excitation causes the species to become electronically excited, often resulting in increased reactivity, which can lead to the formation of non-luminescent by-products. Further to this, a spin-forbidden intersystem crossing might occur due to relaxation of spin-selection rules, causing the fluorophore to become trapped into an excited state of triplet multiplicity. These states are usually very long-lived, because the relaxation to the singlet ground state is also spin forbidden. Unwanted reactions can therefore occur from the long-lived triplet excited state. Lastly, the capacity to discriminate between the probe fluorescence signal and cellular autofluorescence is often problematic, especially if the two emissions occur in similar spectral regions. Cellular autofluorescence originates from endogenous fluorophores; these are biomolecules that contain π-conjugated systems such as the tryptophan amino acid (≈ 400–450 nm), nicotinamide dinucleotide (NAD^+^; ≈ 420–550 nm) and flavin dinucleotide (FAD; ≈ 475–575 nm) [35]. These drawbacks are often overcome by appropriate experimental design and/or instrumental features, but they are limitations nonetheless that at times cannot be avoided. To circumvent these issues, metal-based luminescent probes are emerging tools as alternatives to the use of organic fluorophores [36–39]. Designing and leveraging coordination chemistry to prepare luminescent metal-based compounds is an exciting research proposition, currently explored by many. Within the life sciences, the ability to design organic ligands to selectively coordinate transition metal ions has most frequently been harnessed to detect labile metal ions in biological systems. The literature contains an abundance of information on this topic, which is beyond the scope of this review. However, some prominent examples include “switch-on probes” that become luminescent upon coordination to Fe [40–42], Cu [43–45] or Zn [46, 47], or “emission-shift probes” which exhibit a shift in emission upon coordination to Fe [48], Cu [49] or Zn [50–53]. There are several excellent reviews on luminescent metal probes for the interested reader [54–57].

In addition to providing the capability to detect labile metal ions in biological systems, coordination chemistry can be leveraged to design or “build in” desired photophysical properties. In this respect, the typical photophysical features of luminescent metal-based complexes can be utilised to overcome the drawbacks associated with organic probes. However, it is important to point out that luminescent metal complexes are not necessarily advantageous or superior to organic fluorophores; instead, they can offer an alternative to current imaging tools. They are indeed less explored, and research in this area is expected to result in important development for expanding the optical imaging toolbox beyond the exclusive capability offered by organic fluorophores.

A summary of the main features that characterise the photophysical properties of metal complexes will be presented here. However, the reader is encouraged to consult some of the detailed reviews that have been published in the past years, if in need of a greater level of detail [58–60]. Luminescent metal complexes are characterised by more diverse photophysical properties, which are mainly dependent on the identity of the metal centre and the chemical structure of the ligands. Transition metal complexes bound to π-conjugated ligands can emit from excited states localised within the ligand (π–π*) or involving both the ligand(s) and the metal centre. The latter are known as charge transfer excited states, usually characterised by the promotion of an electron from the *d* orbitals of the metal centre to the empty π* orbitals of the ligands. These states are commonly referred to as metal-to-ligand charge transfer (MLCT) states. Depending on the metal complex, these charge transfer states can also be ligand-to-metal (LMCT), albeit these appear to be less common in the field of luminescent transition metal complexes. Depending on the nature of the conjugated ligand, ligand-to-ligand charge transfer (LLCT) states are also possible. An important feature of heavy metals is their relatively strong spin–orbit coupling, which tends to increase from the first to the third transition periods. A strong spin–orbit coupling favours intersystem crossing, which facilitates the transition from excited states of singlet multiplicity to excited states of triplet multiplicity. Intersystem crossing competes with fluorescent decay, meaning that a strong spin–orbit coupling favours phosphorescent emission, although in some cases a certain degree of fluorescence can still be observed. The emission from phosphorescent metal complexes is therefore characterised by a relatively large Stokes shift. This shift originates from the fact that the intersystem crossing between the singlet and triplet state of the same excited electronic configuration is accompanied by a stabilisation (Hund’s rule) [61]. This stabilisation means that the energy gap between the excited state and ground state is reduced, and hence the emission profile is red-shifted. Another feature of phosphorescent decay is the relatively longer excited state lifetime when compared to fluorescence. This elongation originates from the fact that the transition between the triplet excited state and the singlet ground state is spin-forbidden, and hence it takes longer to occur with respect to the spin-allowed fluorescent transition between a singlet excited state and a singlet ground state. It should be noted though that the spin–orbit coupling of the metal centre also facilitates phosphorescent decay [61].

Along transition metals, lanthanoids have also been widely investigated as luminescent complexes [62]. The photophysical properties of lanthanoid complexes are however distinctly different. The electronic transitions involved in lanthanoid photophysics are intraconfigurational *f–f* transitions. While other transitions are possible, such as *f*–*d* or charge transfer, their discussion goes beyond the scope of this review. *f–f* Transitions are symmetry- and often spin-forbidden, the latter depending on the specific electronic configuration of the lanthanoid element, meaning that trivalent lanthanoids do not absorb photons efficiently. To overcome this issue, the adopted strategy is to coordinate chromophoric π-conjugated ligands to the lanthanoid centre to activate sensitisation via the so-called antenna effect. The conjugated ligand absorbs photons through π–π* electronic transitions, populating singlet excited states. The strong spin–orbit coupling of the lanthanoid centre promotes efficient intersystem crossing, populating the ligand triplet excited state, which then transfers its energy to the lanthanoid centre. Emission from lanthanoid elements spans from the visible (e.g. Eu, Tb) to the near infrared (e.g. Yb, Nd, Er). The emission profile, usually appearing as line-like peaks rather than broad bands, is dependent on the identity of the lanthanoid centre and relatively unaffected by the coordinated ligands (e.g. Eu is always red-emitting, while Tb is always green-emitting), due to the inner core nature of the valence 4*f* electrons. This is very advantageous because structural ligand variations to tune biological properties of the complex can be optimised independently from the photophysical properties, contrary to the general case of transition metal complexes. In common with the luminescence of transition metals, lanthanoid complexes are characterised by large Stokes shifts and relatively long excited lifetime decays. While lanthanoids offer considerable advantages, their labile nature requires the design of ligands with high denticity to confer stability in cellular environments.

The above-listed photophysical characteristics that distinguish metal complexes from organic species can be exploited to alleviate the drawbacks of traditional fluorophores. A large Stokes shift means that concentration quenching phenomena can be minimised and background autofluorescence can be conveniently excluded with the use of appropriate spectral filters. The capability for custom-designing probes with a large Stokes shift can enable multi-modal imaging with direct spectroscopic analyses, such as Raman microscopy, which would otherwise be inhibited by fluorescence overlap with the relatively weak Raman scattering bands [63]. Furthermore, the relatively long excited state lifetime decay is amenable to time-gated microscopy techniques, whereby the emission from the sample is collected after a time-delay from excitation. Since endogenous autofluorescence occurs within few tens of nanoseconds, time gating the signal acquisition ensures the detected emission mostly, if not exclusively, belongs to the probe [64]. The unique photophysical properties of luminescent metal complexes make them suitable for alternative imaging techniques such as super-resolution microscopy. Some notable techniques include stimulated emission depletion (STED) microscopy, stochastic optical reconstruction microscopy (STORM) and structured illumination microscopy (SIM). These techniques might demand more specialised probes but offer superior sub-diffraction resolution. Notable examples which demonstrate applicability with these techniques include Ru(II) complexes and, specifically for lipid imaging, Mn(II) complexes. An alternative technique is two-photon microscopy, where increased imaging depth and reduced phototoxicity are at the expense of decreased resolution in comparison to confocal microscopy. Two-photon excitation requires the simultaneous absorption of two photons with approximately twice the wavelength required for single-photon excitation. The use of longer-wavelength photons reduces photodamage to the sample, and provides greater penetration depth. However, due to the comparatively weak emission process, suitable probes must have high quantum yield, which might reduce the number of suitable metal probes.

The metal centre also offers a multi-modal analytical advantage by providing the ability to detect the metal through techniques such as MRI [65], nanoSIMS [66] and ICP-MS [67, 68]. Although MRI does not typically offer cellular spatial resolution, it is compatible with whole-body or whole-organ imaging in live animals, which can facilitate correlation between biochemical studies at the tissue level in vivo and subsequent subcellular studies ex vivo. Probes containing metal centres have increased electron density associated with the metal centre; therefore, detection with electron microscopies [69, 70] or ion microscopies (nanoSIMS) [71] is possible, which allows for imaging with spatial resolution below 100 nm. Quantification using these microscopies is difficult; however, opportunities for quantitative measurements such as cellular uptake are enabled using ICP-MS or other ICP analysis [67, 68].

Finally, and of most importance, through careful selection of ligands, one can enable differential targeting of the probe to a variety of regions within the cell. Multiple examples already exist in the literature for designing metal-based probes to target specific cellular organelles [72]. More recently, several groups have reported metal-based probes that are selective to lipids or to different lipid species (e.g. neutral vs polar lipids). Metal-based luminescent lipid probes now appear poised to contribute substantially to our understanding of lipid biology and the mechanisms through which lipids promote health or disease. In this review, we examine recent work on the serendipitous discovery or rational development of luminescent metal complexes as lipid probes. These probes have facilitated knowledge advancement of lipid biology and have provided future directions and opportunities for the field of lipid imaging. The review is structured with respect to biological application, specifically describing the use of metal complexes for the study of lipid droplets, phospholipid membranes and lastly generic lipid staining (not specific to any subcellular organelle or compartment). A summary of all the data collected from the literature regarding metal complexes for the imaging of lipids is reported in Table 1. The table is structured to capture a broad overview of the photophysical properties of the complexes and their use in imaging. From a certain point of view, the data highlight the infancy of this research, for which standardised protocols of testing are not yet available. Therefore, probes are often tested on different cell lines, at different concentrations and with various incubation times. For the future development of this research field, some consistency will be required, especially in view of trying to establish structure–activity relationships that will guide an optimisation of metal-based probes based on rational design rather than serendipity.

**Table 1:** Summary of photophysical properties, imaging/staining conditions and biological properties of the complexes

Probe	Metal	Photophysical properties in solution					Imaging and staining conditions					Biological properties			Xlog *P*^a^	References
		λabs (nm)	λem (nm)	Φaer	Τaer	Solvent	Biological staining	Target	λex for imaging (nm)	Staining concentration	Staining media	Uptake mechanism	Cytotoxicity	log *P*		
1	Re	266	569	0.103	2373 (86%) ns, 575 (14%) ns	1% DMSO/water	Live cells, fixed cells, fixed tissue	Lipid droplets	830 (TPE)	10 μM	PBS	–	No signs of cytotoxicity (MTS)	–	1.601	[102]
2	Ir	470	616	0.078	–	MeCN	Living cells, living organism	Lipid droplets	580–670	10 μM	Serum-free media	Energy-dependent pathway	> 40 μM (IC50, 48 h, A549, HeLa, HepG2 and LO2 cells)	2.15	5.249	[67]
3	Ir	470	618	0.056	–	MeCN	Living cells, living organism	Lipid droplets	580–670	10 μM	Serum-free media	–	> 40 μM (IC50, 48 h, A549, HeLa, HepG2 and LO2 cells)	2.19	5.799	[67]
4	Ir	275	612, 664	0.09	0.83 μs	DCM	Live cells	Lipid droplets	405	10 μM	Pluronic F127/water (1/100)	Energy-dependent, mixed caveolae- and clathrin-mediated endocytosis	52.9 ± 3.7 μM (dark) 1.1 ± 0.1 μM (light) (IC50, 24 h, HeLa cells)	1.08 ± 0.05	–	[104]
5	Cu	309	510	0.005	250 (76%) ps, 2.79 (24%) ns	MeCN	Live cells	Lipid droplets	488	25 μM	–	–	–	–	5.915	[105]
6	Zn	390	630	0.44	0.49 (80%), 1.55 (20%) ns	DMSO	Live cells, fixed tissue	Lipid droplets	543, 790 (TPE)	2 μM	DMSO/culture medium (1/250)	Energy-dependent, clathrin-mediated endocytosis	8 μM (CCK-8, 90% cell viability)	1.8 ± 0.1	8.336	[108]
7	Au	256	580	0.045 ± 0.005	0.45 ± 0.02 μs	Hexane	Fixed cells, fixed tissue	Lipid droplets	405, 710 (TPE)	1 mg/mL	Isopropanol/water (1/1)	–	–	–	–	[109]
8	Ru	–	655	0.0044	117	Liposome	–	Phospholipids	440	–	Acetonitrile	–	–	–	5.398	[113]
9	Ru	–	–	–	–	–	Live cells, fixed cells	Phospholipids	488	2 µM	Buffer containing preformed LUVs or calf thymus D	–	–	–	6.830	[125]
10	Ru	–	655	0.0186	155	Liposome	–	Phospholipids	440	–	Acetonitrile	–	–	–	8.262	[113]
11	Ru	–	655	0.011	158	Liposome	–	Phospholipids	440	–	Acetonitrile	–	–	–	11.126	[113]
12	Ru	–	660	0.0031	86	Liposome	–	Phospholipids	–	–	–	–	–	–	6.003	[113]
13	Ru	–	660	0.0073	97	Liposome	–	Phospholipids	440	–	Acetonitrile	–	–	–	4.571	[113]
14	Ru	–	660	0.0104	105	Liposome	–	Phospholipids	440	–	Acetonitrile	–	–	–	7.435	[113]
15	Ru	–	–	–	–	–	–	Phospholipids	–	–	–	–	–	–	8.422	[114]
16	Ru	285	655	0.027	368 ns	Liposome in PBS	–	Phospholipids	–	–	–	–	–	–	7.258	[115]
17	Ir	250	600	0.084	610, 161, 70 ns	Liposome in PBS	–	Phospholipids	–	–	–	–	–	–	10.654	[115]
18	Pt	ca. 375	528	0.30	1.95 μs	HEPES buffer	Live cells	Phospholipids	400, 720 (TPE)	1 μg/mL	Culture media	–	10 μg/mL HeLa cells, ca. 90% cell viability, 25 h 10 μg/mL epithelial cells, ca. 70% cell viability 25 h	–	14.303	[116]
19	Ru	258	613	–	0.68 ± 0.017 μs	9/1 MeCN/DMSO	Live cells	Phospholipids	458	35 μM	Culture media	–	35 μM SP2 myeloma cells, ca. 70% cell viability	–	5.129	[63]
20	Ru	258	614	–	0.78 ± 0.022 μs	9/1 MeCN/DMSO	Live cells	Phospholipids	458	35 μM	Culture media	–	35 μM SP2 myeloma cells, ca. 70% cell viability	–	6.674	[63]
21	Re	380	550	–	–	–	Live cells	Phospholipids	405	100 μg/mL	–	–	–	–	1.343	[118]
22	Re	380	550	–	–	–	Live cells	Phospholipids	405	100 μg/mL	–	–	100 μg/mL MFC-7 cells, ca. 25% cell viability	–	3.491	[118]
23	Re	380	550	–	–	–	Live cells	Phospholipids	405	100 μg/mL	–	–	–	–	4.923	[118]
24	Ir	ca. 295	587	0.18	0.68 μs	DCM	Live cells	Phospholipids	488	5 μM	–	Energy-dependent, endocytosis	–	9.89	13.300	[68]
25	Al	394	643	0.42	4.3 ns	DMSO	Live cells	Phospholipids	561	1 μM	DMSO/culture medium (1/2000)	Membrane potential-dependent passive diffusion	2 μM HeLa cells, ca. 90% cell viability, 48 h	–	7.174	[119]
26	Tb	–	–	–	–	–	Live cells	Phospholipids	–	–	–	–	–	–	–	[120]
27	Ir	263	618	0.055	131 ns	DCM	Live cells	General lipophilic staining	403	20 μM	DMSO/culture medium (1/500)	Energy dependent pathway	Low (40 μM, 24 h) (MTS)	2.09 ± 0.06	3.904	[68]
28	Ir	256	542	0.042	140 ns	DCM	Live cells	General lipophilic staining	403	20 μM	DMSO/culture medium (1/500)	Energy dependent pathway	Low (40 μM, 24 h) (MTS)	2.01 ± 0.05	4.548	[68]
29	Ir	268	560	0.023	114 ns	DCM	Live cells	General lipophilic staining	403	20 μM	DMSO/culture medium (1/500)	Energy dependent pathway	Low (40 μM, 24 h) (MTS)	2.68 ± 0.08	5.8444	[68]
30	Ir	263	580	0.021	153 ns	DCM	Live cells	General lipophilic staining	403	20 μM	DMSO/culture medium (1/500)	Energy dependent pathway	Low (40 μM, 24 h) (MTS)	2.23 ± 0.04	5.484	[68]
31	Ir	260	575	0.036	497 ns	DCM	Live cells	General lipophilic staining	403	20 μM	DMSO/culture medium (1/500)	Energy dependent pathway	Low (40 μM, 24 h) (MTS)	2.57 ± 0.05	5.484	[68]
32	Ir	257	635	0.028	218 ns	DCM	Live cells	General lipophilic staining	403	20 μM	DMSO/culture medium (1/500)	Energy dependent pathway	High (40 μM, 24 h) (MTS)	1.68 ± 0.05	3.985	[68]
33	Ir	–	520	0.062	332 (88%) ns, 38 (12%)	0.1% DMSO/water	Live bacteria	General lipophilic staining	405	20 μM	DMSO/culture media (1/1000)	–	Low	–	4.907	[66]
34	Ir	–	552	0.057	628 (59%) ns, 189 (41%) ns	0.1% DMSO/water	Live bacteria	General lipophilic staining	405	20 μM	DMSO/culture media (1/1000)	–	Low	–	4.548	[66]
35	Ir	–	600	0.039	928 (75%) ns, 305 (25%) ns	0.1% DMSO/water	Live bacteria	General lipophilic staining	405	20 μM	DMSO/culture media (1/1000)	–	Low	–	6.128	[66]
36	Re	276	517	0.25	5 (57%) ns, 10 (43%) ns	DCM	Live bacteria, fixed bacteria	General lipophilic staining	–	–	–	–	–	–	4.963	[71]
37	Pt	282	515	0.28	11 ns	DCM	Live bacteria, fixed bacteria	General lipophilic staining	488	10 μM	DMSO/nutrient broth (1/1000)	–	–	–	10.580	[71]
38	Pd	256	585, 638	0.12	0.88 ns	Cyclohexane	–	General lipophilic staining	–	–	–	–	–	–	4.978	[121]
39	Pd	258	595, 650	0.50	4.46 ns	Cyclohexane	–	General lipophilic staining	–	–	–	–	–	–	6.376	[121]
40	Pd	253	587, 636	0.14	1.05 ns	Cyclohexane	–	General lipophilic staining	–	–	–	–	–	–	8.406	[121]
41	Pd	760	660	–	–	5% DMSO/water	Live cells, living organism	General lipophilic staining	561, 760 (TPE)	5 μM	DMSO/culture medium (1/200)	–	Low (< 20 mM) (MTT)	–	8.839	[123]
42	Cr	337	395, 415	–	0.2 (78.5%) ns, 6.0 (21.5%) ns	DCM	Live cells	General lipophilic staining	405, 810 (TPE)	50 μM	Ethanol/culture medium (1/50)	–	100 μM ca. 10% viability 4 h (Alamar Blue) and 15 min (LDH)	–	8.153	[124]
43	Mn	455	480	0.29	4.51 ns	DCM	Live cells, fixed tissue	General lipophilic staining	405, 760 (TPE)	5–10 μM	–	–	Low (80 μM, 24 h) (MTT)	–	12.560	[65]

^a^Values calculated using the Xlog*P* v2.0 method

## Discussion

Phosphorescent metal complexes can be broadly divided into four classes. Hexacoordinated second- and third-row transition metal complexes of n*d*^6^ electronic valence configuration are amongst the most popular. Classical examples for this type of complexes are Re(I) tricarbonyl diimine [73–76], Ru(II)/Os(II) polypyridine [77–80] and cyclometalated Ir(III) complexes [81–83]. These complexes have received a great deal of focus in the past decades for their application both as diagnostic and therapeutic agents. The latter application can exploit photoactivated ligand exchange reactions as well as photo-generation of toxic reactive oxygen species such as ^1^O_2_ within cells [84]. A second class of metal-based luminophores is represented by complexes of Pt(II), Au(III) and Au(I) [85–89]. For this class, most of the effort has been centred around Pt(II) complexes; however, complexes of Au are starting to emerge as promising candidates for imaging. The photophysical properties of these complexes are well known, and these species usually emit from metal-to-ligand charge transfer states of triplet multiplicity (^3^MLCT) or metal-perturbed π–π* ligand-centred emission (^3^LC). This knowledge allows for fine-tuning of their photophysical and photochemical properties to optimise their use in life sciences. Complexes of first-row transition metals are usually non-luminescent, aside from tetrahedral complexes of Cu(I). The lack of luminescence is attributed to low-lying ligand field excited states (^3^LF) that are non-emissive. While paradigm shifts in the use of first-row transition metal luminophores have occurred very recently, these developments are still too young to have found tangible applications in the life sciences [90, 91]. However, examples of lipid-based probes that contain metals such as Cr, Mn, Cu and Zn have been reported and will be discussed in this review. The final class of metal-based probes is represented by lanthanoid complexes [92–96].

Metal-based complexes can be conjugated to biological vectors to impart cellular specificity [77, 97]. Alternatively, their chemical nature can be fine-tuned in order to define biological properties such as membrane permeability, organelle localisation and/or interaction with specific biomolecules. There are currently rules of thumb available for the design of probes to target various regions of the cell, and these usually rely on charge and lipophilicity. The charge is important for solubility in aqueous medium, which is essential during the incubation stage, and to favour internalisation by exploiting membrane potentials. Many investigated probes are cationic in nature, albeit more recently neutral probes have started to gain attention due to the fact that their biological properties and cytotoxicity might be fundamentally different. The lipophilicity also can assist during internalisation, for example by favouring passive diffusion through the cell membrane, and preferential interaction with specific organelles.

### Lipid Droplets

Lipid droplets (LDs) are cytoplasmic organelles that store excess lipids such as sterol esters and triacylglycerols [98]. Their exterior consists of a phospholipid monolayer, and depending on droplet depletion or growth, metabolic intermediates such as fatty acids and unesterified sterols [99]. At their core, LDs contain neutral lipids such as triglycerides and cholesterol esters [100]. LDs and their associated processes are vital for lipid homeostasis, and as such, LDs have been implicated in conditions such as type 2 diabetes, cardiovascular disease, Alzheimer’s disease and a variety of cancers, which all exhibit lipid imbalances and alterations during their progression [101]. Therefore, it is critical to have accessible methods to study LD composition and their roles in lipid homeostasis.

Targeting LDs can be achieved with lipophilic probes that are able to penetrate cells, where localisation within the neutral core of LDs occurs via hydrophobic interactions. Probes which target LDs have log*P* values spanning a large range, between 1.5 and 8.5, with a general trend of high-lipophilicity probes displaying specificity for LDs due to localisation within the core of LDs. As lipophilicity decreases, specificity to LDs is reduced, and more diffuse localisation with targeting of other organelles is observed. On the other hand, highly lipophilic compounds are characteristically difficult to solubilise in culture media and often require the use of cytotoxic solvents (such as dimethyl sulfoxide [DMSO], acetonitrile or ethanol), which can damage the cell membrane and produce misleading results.

#### Imaging Lipid Droplets with Metal-Based Probes

##### Probes Incorporating Third‑Row Transition Metals (Ir, Re)

**Fig. 2:**
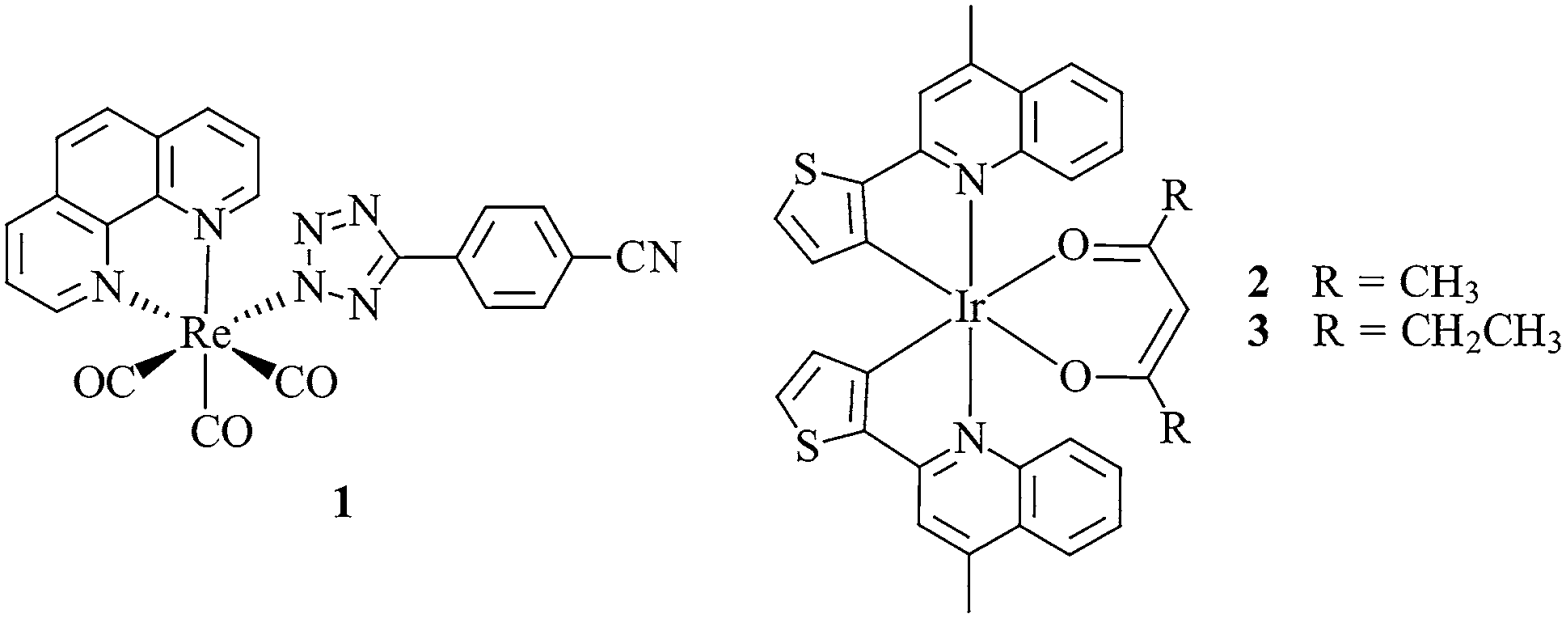
Chemical structures of **1**–**3**

In 2014, the luminescent metal complex **1** was reported by Bader et al. to stain LDs (Fig. 2) [102]. The neutral rhenium(I) tricarbonyl 1,10-phenanthroline complex bound to 4-cyanophenyltetrazolate was shown to localise in regions with high concentrations of polar lipids such as phosphatidylethanolamine, sphingomyelin, sphingosine and lysophosphatidic acid in the LDs of mammalian adipocytes [103]. The low toxicity combined with the high photostability of **1** enabled its use for live imaging of *Drosophila* larval adipose tissues. The moderately lipophilic probe revealed lipid distribution when imaged by confocal microscopy, displaying higher affinity for polar lipids at the edges of the LDs compared to neutral lipids of the LD core. Correlative imaging using Raman and FTIR microscopy confirmed this finding, where a strong correlation between the probe and polar lipids of the LDs was shown.

Two lipophilic neutral cyclometalated Ir(III) complexes, **2** and **3**, have shown to be suitable probes for LD visualisation in live cells and organisms [67]. When applied to A549 cells and co-stained with BODIPY 493/503, significant co-localisation was observed, confirming LD accumulation. Featuring large two-photon absorption cross sections and long excited state lifetimes, the suitability of these probes for two-photon phosphorescence lifetime imaging microscopy (PLIM) was demonstrated, allowing for the elimination of background autofluorescence. Optical imaging followed by ICP-MS measurements reveals higher uptake efficiency of **2** in comparison to **3**. Complex **2** was shown to be compatible with living organisms such as zebrafish for the visualisation of lipid metabolism in vivo using optical imaging.

**Fig. 3:**
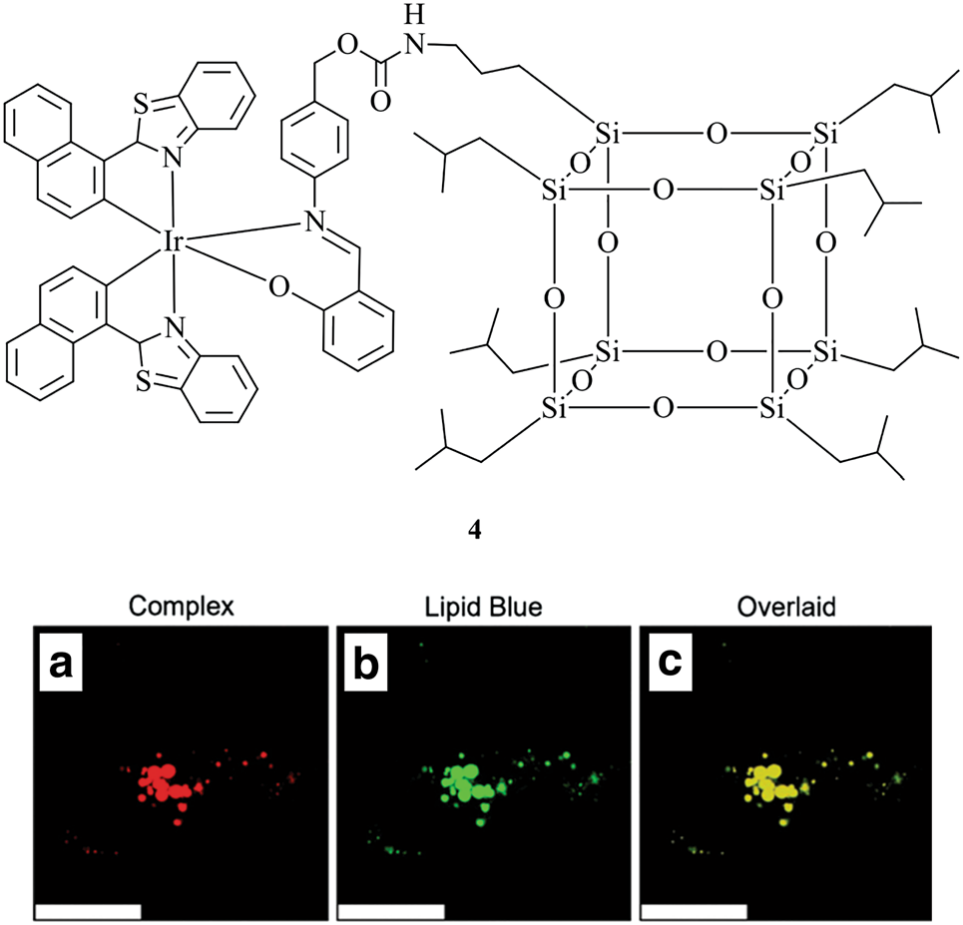
Chemical structure of **4**. Bottom panel shows **4** (red) (**a**) incubated in 3T3-L1 differentiated adipocytes and co-stained with Lipid Blue (green) (**b**) and merged image (**c**). Scale bar: 25 μm. Reprinted (adapted) with permission from Zhu et al. [104]. Copyright 2021 American Chemical Society

Probe **4** is another example of a neutral cyclometalated Ir(III) complex that is specific to LDs (Fig. 3) [104]. The probe features a bulky polyhedral oligomeric silsesquioxane (POSS) cage, which, when compared to the POSS-free counterpart, has a higher lipophilicity (experimentally determined log*P* = 1.08 and 0.63, respectively). When applied to HeLa cells, the POSS unit showed unaltered specificity to lipid droplets, which is demonstrated by co-localisation studies using Lipid Blue. However, **4** has low water solubility, requiring the use of a triblock copolymer to make a solid dispersion in aqueous solution for cell incubation. Despite the addition of the bulky POSS unit, **4** retains high cell uptake via energy-dependent pathways. The attachment of the POSS unit resulted in a reduced cytotoxicity of the complex in the dark, but its phototoxicity upon radiation provides potential use in photodynamic therapy. Complex **4** was applied to cancer cells such as Neuro-2a, a fast-growing neuroblastoma cell line, revealing significantly increased emission from LDs in comparison to normal cells under the same experimental conditions.

##### Probes Incorporating First-Row Transition Metals (Cu, Zn)

**Fig. 4:**
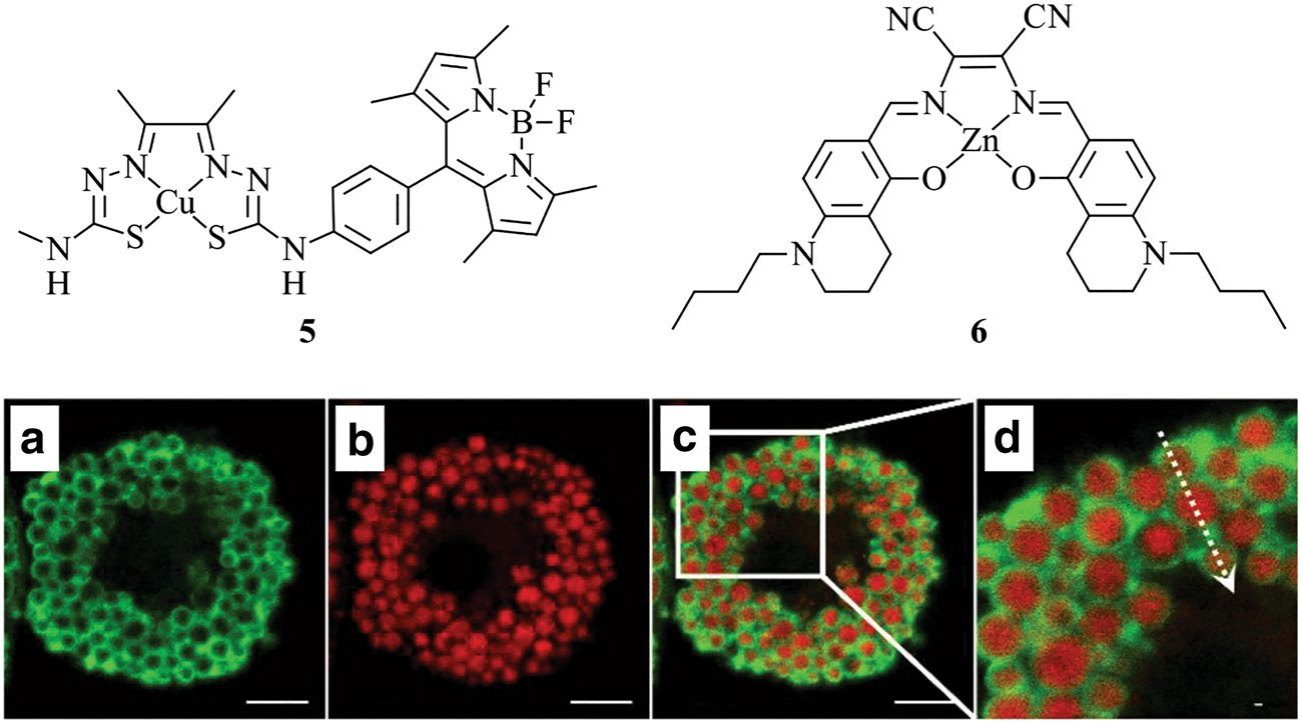
Chemical structures of **5**–**6**. Analysis of adipocytes with immunofluorescence (green, **a**) and uptake of compound **5** (red, **b**), merged images of (**a**, **b**) (**c**, **d**). Reproduced with permission from Tang et al. [108]

Hickey et al. report the synthesis of probe **5**, a BODIPY-labelled copper bis(thiosemicarbonato) complex (Fig. 4) [105]. These complexes are generally weakly fluorescent, but have gained interest due to their potential neuroprotective capability [106]. Enabling the detection of this probe often requires the inclusion of a fluorescent tag [107] such as BODIPY, providing **5** with high quantum yield and high stability under physiological conditions. Fluorescence lifetime imaging microscopy (FLIM) is enabled due to the difference in emission lifetime of **5** compared to the copper-free ligand. It is shown that the probe localises within lysosomes of neuronal cells and both LDs and lysosomes in secondary M17 neuroblastoma cells. Once accumulated within LDs, FLIM reveals that some Cu(II) ions are released and the liberated ligand coordinates to bioavailable Zn(II).

Probe **6** (Fig. 4) features Zn(II) bound by a salen (*N*,*N*′-bis(salicylidene)ethylenediamine) ligand, a non-fluorescent complex in an aqueous environment with significant increase of fluorescence in lipophilic environments [108]. Low cytotoxicity and high photostability is reported, enabling its imaging in living adipose cells. Immunofluorescence using perilipin-1 antibodies co-stained with probe **6** in HeLa cells shows that **6** specifically localises within the hydrophobic core of LDs, and co-staining with BODIPY 493/503 shows comparatively higher specificity of **6** for LDs. This behaviour is surprising when considering the reported log*P* value is 1.8; the general trend would anticipate non-specific staining of LDs and polar lipids. Interestingly, when the log*P* is calculated, a value of 8.3 is obtained, which is consistent with the trend, but there is a large discrepancy with the experimental value. The suitability to image LDs stained with **5** in tissues is demonstrated in rat subcutaneous adipose tissue incubated with the probe and subsequent imaging by two-photon microscopy to reveal LDs. Further, the potential for tracking LD biogenesis during adipogenesis of rat preadipocytes is demonstrated, indicating potential for further understanding of LD homeostasis.

##### Metal Clusters

**Fig. 5:**
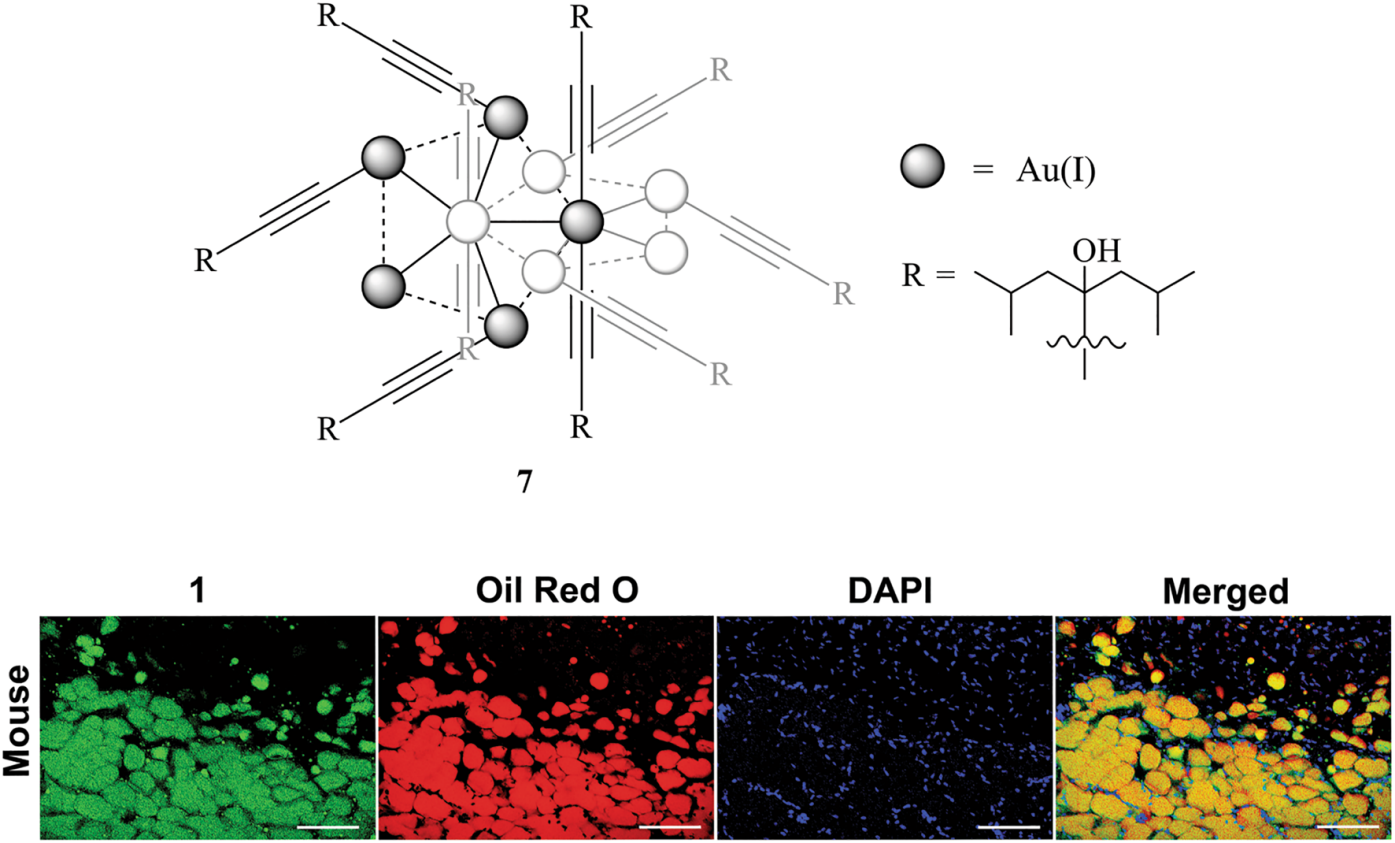
Chemical structure of **7**. The decanuclear molecule contains two interlocked, perpendicular 5-membered rings. The ring which is perpendicular to the page has been greyed out for clarity. Bottom panel shows co-localisation of Oil Red O (red) with **7** (green), counterstained with DAPI (blue) n subcutaneous adipose tissues of mouse. Scale bars: 100 μm [109]. Reprinted (adapted) with permission from Koshevoy et al. [110]. Copyright 2021 American Chemical Society

Mononuclear metal complexes used for biological imaging have received criticism due to being prone to quenching by oxygen and/or water, both which are unavoidable in biological systems [109]. Koshel et al. have synthesised polynuclear homo- and heterometallic gold–alkynyl clusters, such as **7**, which are shown to be insensitive to oxygen and water quenching, while maintaining the favourable spectral properties of mononuclear metal complexes (Fig. 5) [110]. The clusters were evaluated in a broad range of biological applications including various adipose tissues from a range of animals (mouse, chicken, pigeon) as well as Hep G2 cells. The samples were counterstained with DAPI and Oil Red O before being imaged by two-photon microscopy. Despite having a relatively high molecular size, **7** is able to penetrate cells to reveal high affinity to LDs in several tissue types. The suitability of the probe for phosphorescence lifetime imaging is demonstrated in chicken subcutaneous adipose tissues, allowing for the elimination of background signal.

### Phospholipids

Phospholipids are the most abundant lipids comprising the bilayer structure responsible for cell membranes [111]. Additionally, phospholipids also facilitate signalling and energy storage. Phospholipids have two hydrophobic chains that are joined at a polar head. Variations in chain length, saturation and incorporation of cholesterol create alterations in molecular packing and consequently alter the fluidity of the membrane. Mimicking these structural features in the design of probes has been successful in furthering the understanding of this complex relationship. Typically, probes targeting phospholipids incorporate long hydrophobic alkyl chains appended to charged metal complexes, favouring the incorporation of the probe within the lipids of the bilayer. Probes with high affinity for membranes are unlikely to diffuse across the plasma membrane; however, if the probe is internalised via energy-dependent pathways, other lipids and organelles within the cell can be targeted, such as the intracellular membranes associated with the Golgi apparatus, ER and lysosomes.

Bolstered by advancements in phospholipidomics, new evidence indicates that changes in phospholipid composition, metabolism and distribution within tissues, cells and fluids are linked to diabetes, cancer and cardiovascular diseases [112]. To further explore these links, visualisation methods are vital to study phospholipids and uncover their connection to these conditions.

#### Imaging Phospholipids with Metal-Based Probes

##### Probes Incorporating Second- and Third-Row Transition Metals (Ir, Re, Ru, Pt)

**Fig. 6:**
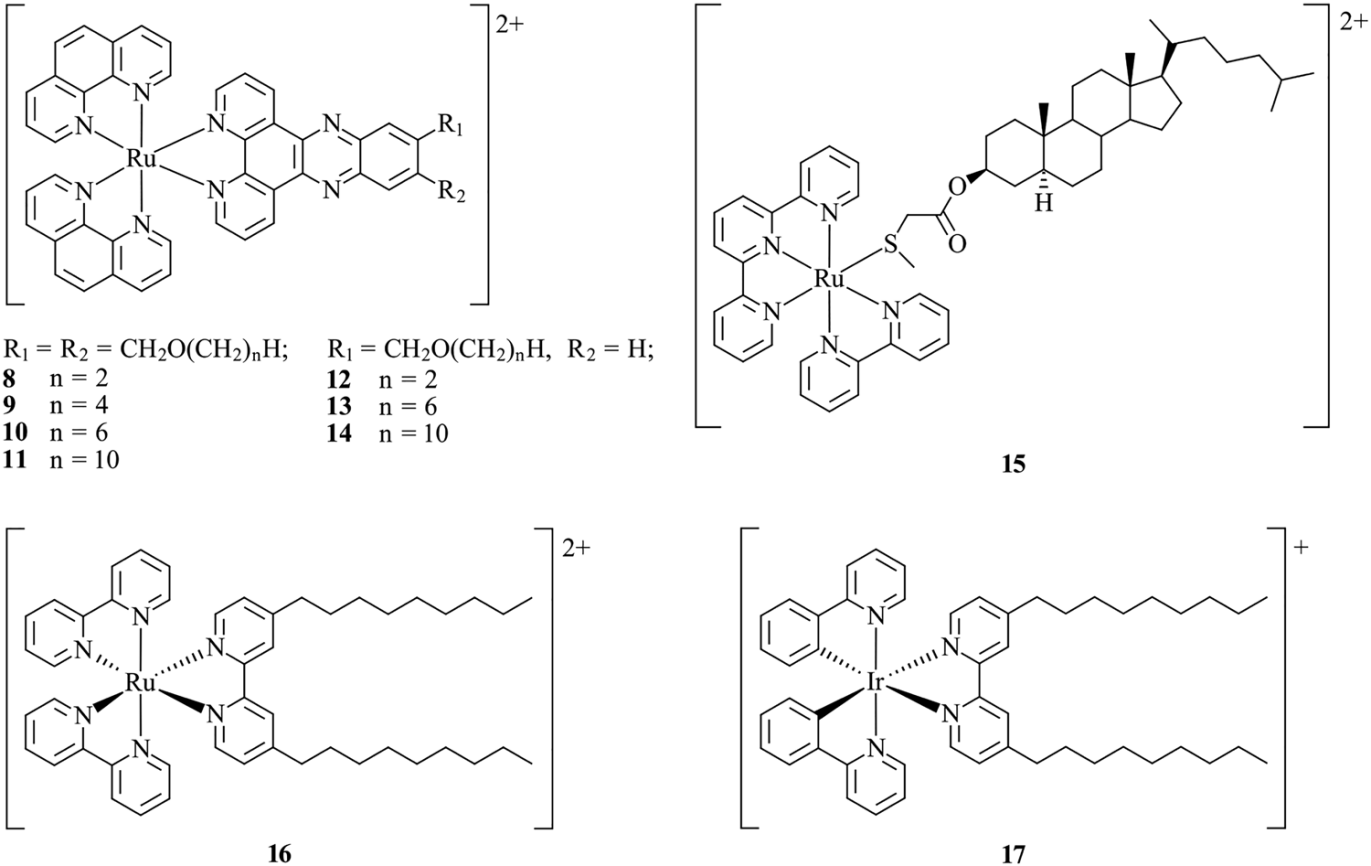
Chemical structures of **8**–**17**

The targeting of lipid bilayers such as the plasma membrane employs the use of ligands with long alkyl chains bound to cationic metal complexes. Such probes based on cationic Ru(II) complexes have been investigated in liposomes for their interactions with phospholipid bilayers in order to further understand the structure and dynamics of lipid membranes (Fig. 6). Ru(II) bound to dipyridophenazine-type ligands **8**–**14** feature varying alkyl chain lengths (*n* = 2, 4, 6, 10), and the number of alkyl chains (one or two) was investigated using flow linear dichroism with phospholipid vesicles as membrane models [113]. Probe **13**, with alkyl chain length of six C atoms, was found to be optimal where the alkyl chain inserts parallel to the lipid chain of the liposome bilayer, and the charged Ru(II) acts as a polar head group.

Bonnet et al. [114] report the synthesis of **15**, a thioether–cholestanol hybrid ligand coordinated to Ru(II) via its sulfur atom (Fig. 6). Complex **15** was applied to liposomes containing negatively charged lipids. Photophysical measurements and cryo-TEM reveal that electrostatic interactions of the probe as well as the insertion of the nonpolar tail are involved in the probe–membrane interaction. Cleavage of the Ru–S bond can be triggered by irradiation, causing the Ru(II) complex to diffuse away. The authors conclude that this may be a useful method for drug delivery. Mechler et al. [115] synthesise Ru(II) and Ir(III) lipid-mimetic surfactants **16** and **17** (Fig. 6), which are designed to more closely mimic the phospholipid membrane, avoiding the alteration of its biophysical properties. Complexes **16** and **17** were applied to dimyristoyl-phosphatidylcholine liposomes and imaged by confocal microscopy, highlighting membrane localisation. Small-angle X-ray scattering (SAXS) measurements and atomic force microscopy (AFM) imaging are used to demonstrate that the bilayer is unaltered by the addition of the probes. The mimetic approach is demonstrated as an effective tool for biophysical characterisation of lipid membranes.

**Fig. 7:**
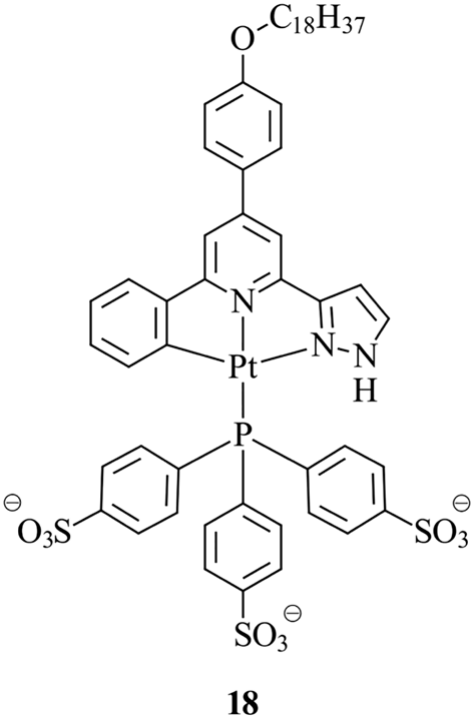
Chemical structure of **18**

A water-soluble and amphiphilic Pt(II) complex, **18**, has been developed by Koo et al. [116] for in vitro plasma membrane staining (Fig. 7). The complex features a trisulfonated triphenylphosphine bound to the Pt(II) for enhancement of water solubility, and a C18 alkyl chain on the cyclometalated ligand to increase lipophilicity. Two-photon confocal microscopy of the complex incubated within live HeLa cells reveals exclusive plasma membrane staining, observing no subcellular migration after 3 h. The poor cellular uptake and plasma membrane specificity may be rationalised by the negatively charged sulfonate groups, which inhibit internalisation. On the other hand, the long hydrophobic alkyl chain favourably interacts with the membrane.

**Fig. 8:**
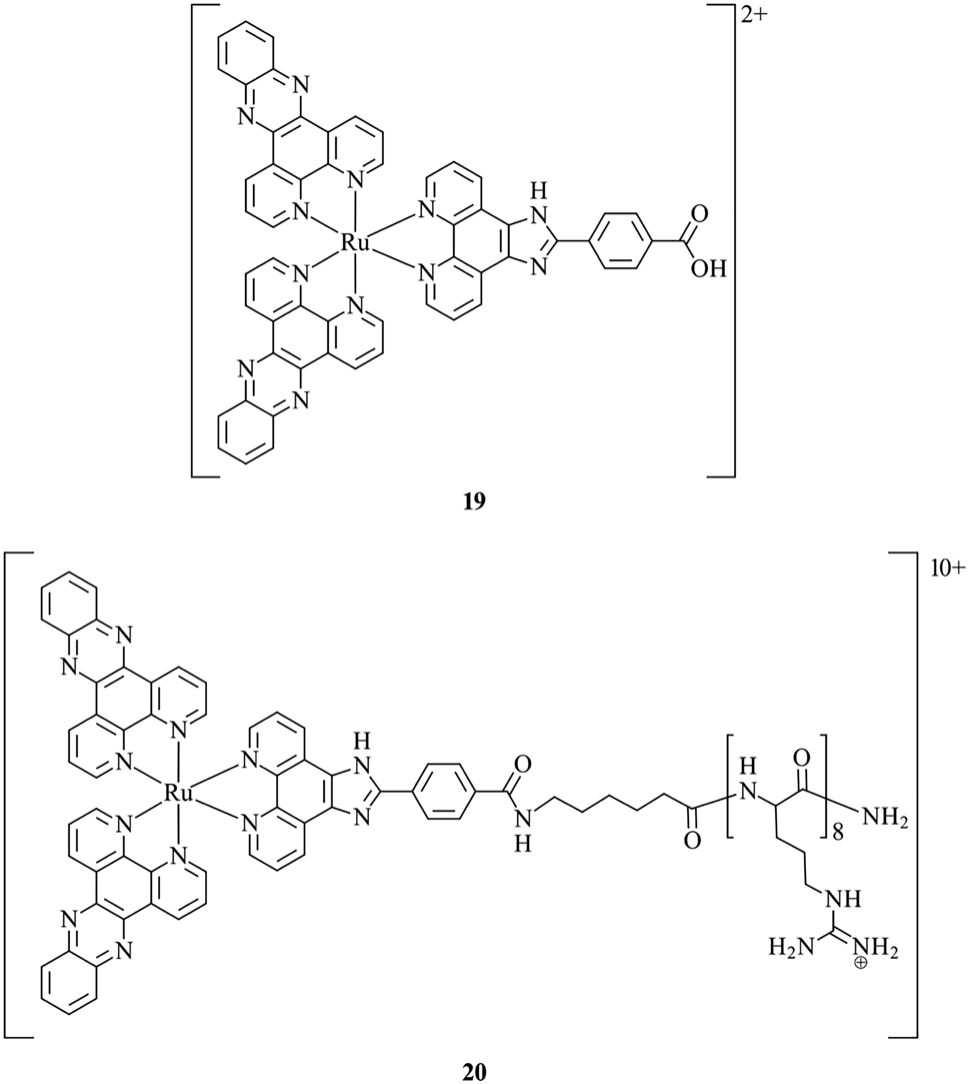
Chemical structures of **19**–**20**

Cosgrave et al. [63] report probes **19** and **20**, which are Ru(II) complexes featuring dipyridophenazine ligands and an ancillary 2-(4-carboxyphenyl)-1H-imidazo[4,5-*f*][1,10]phenanthroline ligand (Fig. 8). When applied to live SP2 myeloma cells, **19** does not enter the cell, and exclusive staining of the outer cell membrane is observed. However, **20** includes a cell-penetrating polyarginine peptide which is shown to facilitate passage into the cell via endocytosis, resulting in internal membrane staining of subcellular structures. Detection of the probe using resonance Raman spectroscopy is enabled due to its large Stokes shift, whereby an interference-free resonance Raman spectra can be collected. Applying this technique to live myeloma cells validates the localisation of **19** and **20** within the cell membranes. In a more recent study, **20** was utilised as the first example of a metal complex for STED microscopy, owing to the high photostability, red-shifted emission, and low cytotoxicity [117]. By performing STED imaging of HeLa cells, **20** highlights the tubular structure of the ER with high resolution and with improved performance in comparison to currently used organic fluorophores. This example demonstrates the use of cell-penetrating peptides as a means to enhance internalisation for those metal complexes that display poor cellular uptake.

**Fig. 9:**
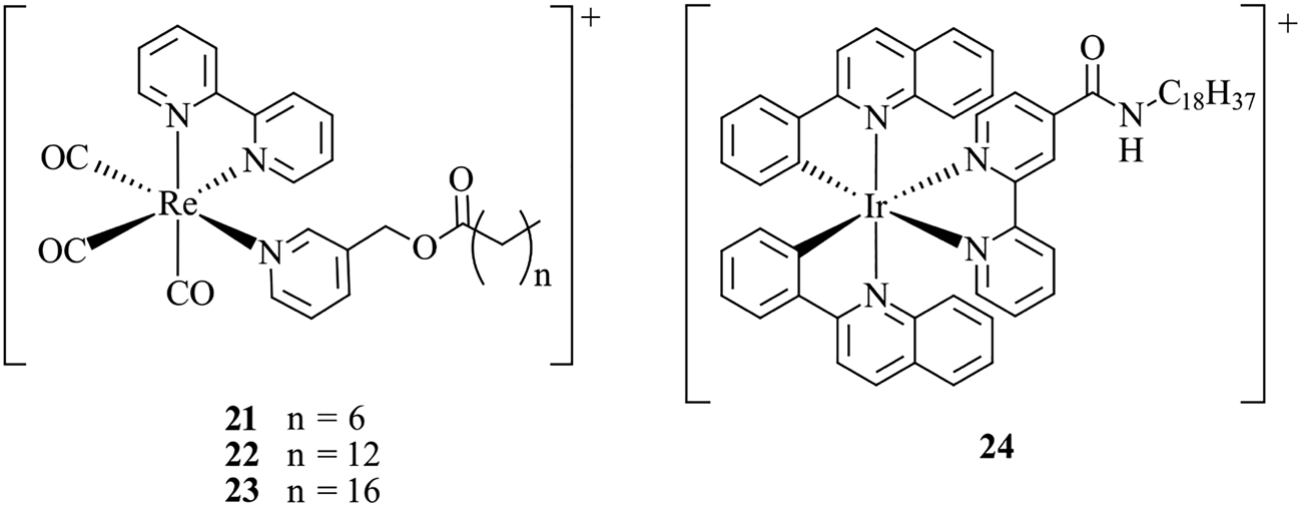
Chemical structures of **21**–**24**

Fernández-Moreira et al. [118] report a series of cationic Re(I) probes, **21**–**23**, with varying alkyl chain lengths (*n* = 6, 12, 16) (Fig. 9). When applied to MCF-7 cells, the shorter chain analogues, **21** and **22**, show general cytoplasmic staining, whereas the complex with the longest chain (*n* = 16), **23**, shows high membrane specificity. Lo et al. [69] report a series of lipophilic Ir(III) complexes, which were applied to artificial biological membranes consisting of DSPC (1,2-distearoyl-*sn*-glycero-3-phosphocholine). The most notable probe is **24**, a cationic Ir(III) complex containing a C18 alkyl chain (Fig. 9). The probe was shown to incorporate within the liposomes by cryo-TEM imaging and photophysical studies, revealing that **24** is embedded within the hydrophobic region of the phospholipid DSPC. Interestingly, when **24** is incubated in HeLa cells, uptake via an energy-dependent pathway results in general lipophilic staining rather than exclusive membrane staining. The authors propose that the complex is likely interacting with the lipid-rich regions of the endoplasmic reticulum, mitochondria and Golgi apparatus. These examples nicely illustrate the link between functionalisation with alkyl chains of variable lengths, cellular internalisation and specificity.

##### Probes Incorporating p‑Block Metals (Al)

**Fig. 10:**
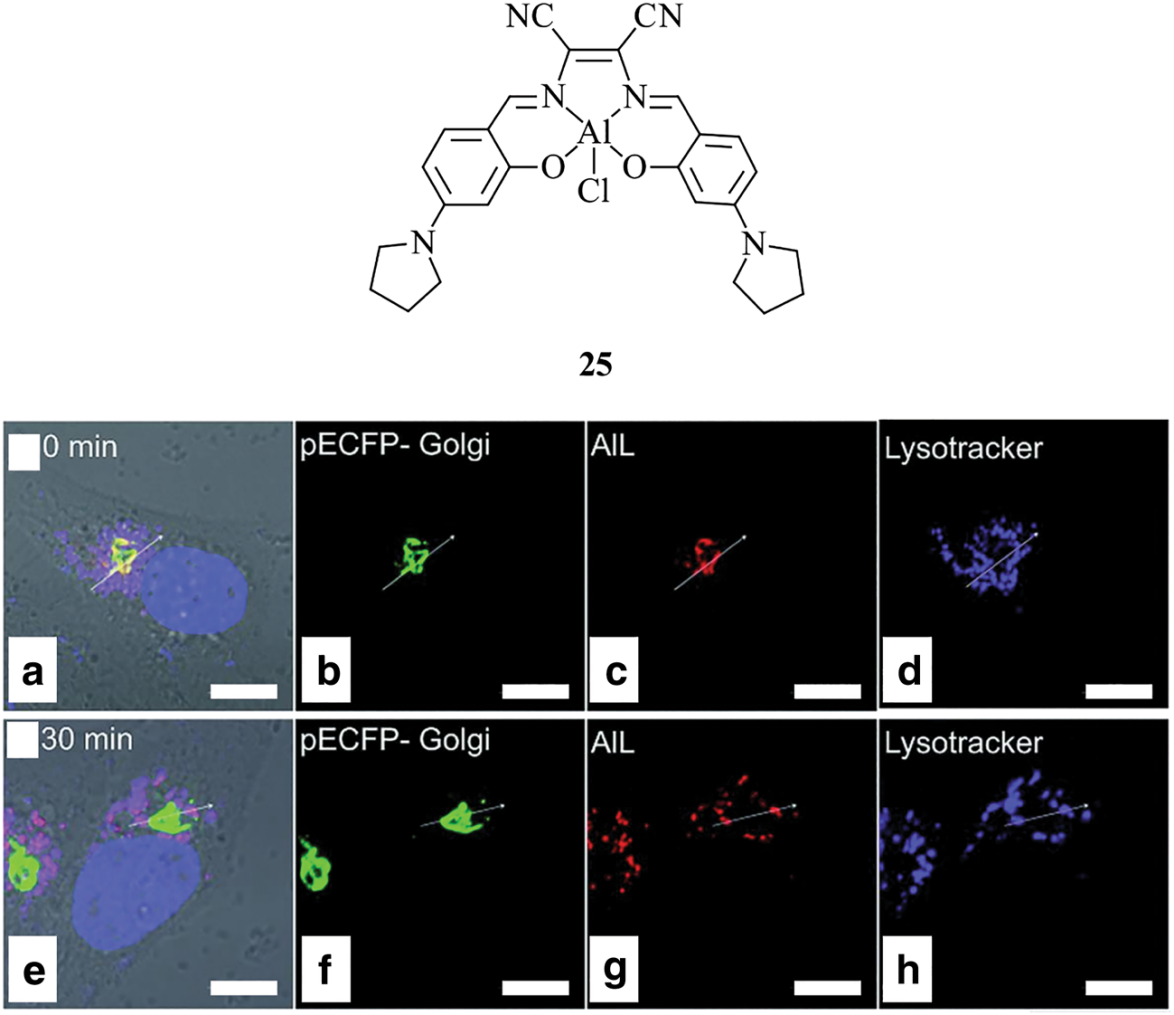
Chemical structure of **25**. Bottom panel showing merged brightfield images of HeLa cells, incubated with (**a**, **e**) of pECFP-Golgi (green) (**b**, **f**), **25** (red) (**c**, **g**) and LysoTracker^®^ Deep Red (blue) (**d**, **h**) at 0 min (**a**–**d**) and 30 min (**e**–**h**) [119]. Scale bar = 10 μm. Figure reproduced from Tang et al. [119]. Under the Creative Commons Attribution 3.0 Unported Licence

A salen ligand coordinated to Al(III), **25**, has been developed as a probe with selectivity towards phospholipids (Fig. 10) [119]. The selective binding of **25** to various phospholipids was investigated, revealing the Al(III) complex covalently binds to mono-negatively charged and oxo-containing lipids, likely due to the hard Lewis acidity and high oxophilicity of Al(III). This specificity is further demonstrated by the addition of the probe to an artificial fluorescent liposome, whereby the green fluorescence of the liposome showed strong correlation with the red fluorescence of **25**. When applied to living HeLa cells, it was found that the probe binds to the phospholipids of the Golgi apparatus before it is transported to the lysosomes via membrane vesicle trafficking. The potential of **25** as an alternative to the use of fluorescent proteins is highlighted, specifically as a tool for the investigation of the vesicle trafficking from the Golgi apparatus to the lysosomes.

##### Probes Incorporating Lanthanoids (Tb)

**Fig. 11:**
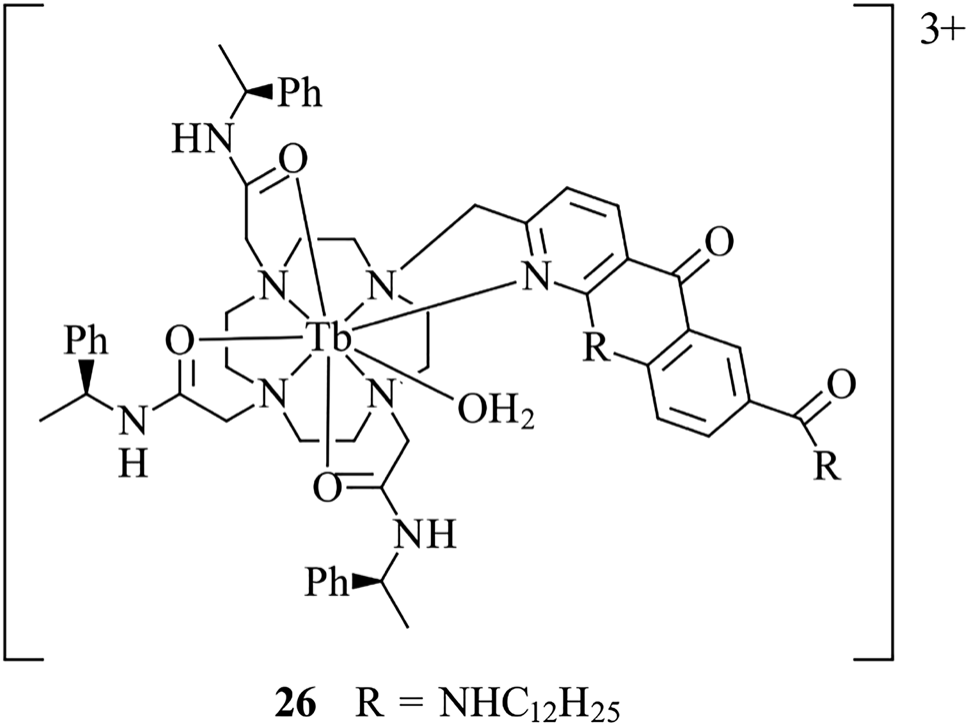
Chemical structure of **26**

An emissive Tb(III) complex, **26**, featuring an azaxanthone chromophore as the light-harvesting group featuring a dodecylamine side chain has been investigated for applications in live cell imaging (Fig. 11) [120]. It was found that the complex is cytotoxic when applied to NIH-3T3 cells, which was ascribed to a disruption of membrane integrity. This feature hinders the application of the probe in live NIH-3T3 cell studies. However, lower cytotoxicity in CHO cells was observed, indicating the potential use as a general membrane stain. This example, along with previous ones, highlights the importance of assessing the properties of molecular probes in multiple cell lines, rather than drawing conclusions on structure–activity relationships after incubation within a single cell type.

### General Lipophilic Compounds

The design of lipid-targeting metal complexes often results in non-specific lipid binding. While lack of specificity might be regarded as a negative feature, non-specific lipid staining is desirable for studying biochemical processes in injury and disease. Non-specific lipid staining is useful to contrast lipidic alterations with cellular processes. Organic probes such as BODIPY [30] and Nile Red [31] have been used to measure bulk lipid alterations in brain white matter in traumatic brain injuries and multiple sclerosis. However, the emission of these compounds occupies a populated area of the spectrum and are not always suited for specific applications due to emission overlap with autofluorescence. Therefore, a platform of molecular probes displaying non-specific lipid binding with tunable emission across the entire visible range would be highly desirable.

#### Staining of General Lipophilic Compounds

##### Probes Incorporating Second‑ and Third‑Row Transition Metals (Ir, Re, Pt, Pd)

**Fig. 12:**
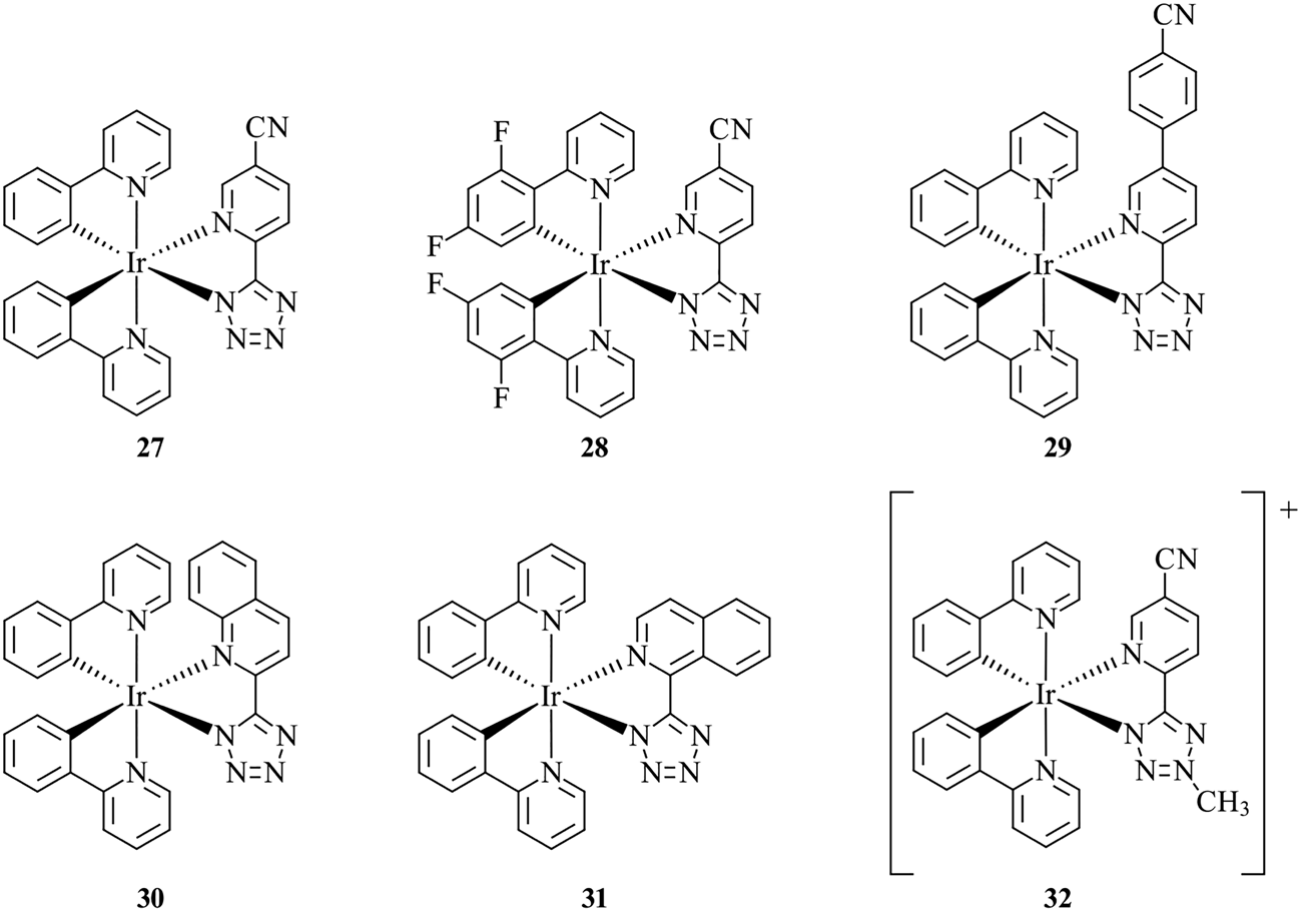
Chemical structures of **27**–**32**

While trends in localisation of probes are observed based on physical properties such as lipophilicity and charge, these properties alone are not enough to rationalise the behaviour when applied to complex systems such as cells and tissues. Caporale et al. report the synthesis and biological investigation of probes **27–32** (Fig. 12), a family of neutral cyclometalated Ir(III) tetrazolato complexes, as well as their methylated cationic analogues [68]. It was found that variations to lipophilicity and charge by altering the cyclometalated phenylpyridine and tetrazolato ligands do not follow expected trends in localisation or uptake behaviour in live cells. For example, the measurement of cellular uptake by ICP-MS shows comparable uptake of the positively charged probe, **32**, in comparison to the neutral probe, **30**, whereas **27** shows a significantly lower uptake. When applied to live H9c2 cells, two-photon confocal microscopy reveals that the complexes **27**–**32** co-stained with BODIPY 500/510 or ER-Tracker display both LD and ER localisation. Comparing the experimentally determined log*P* of **27** and **30**, 2.09 and 2.23, respectively, it would be expected that the higher log*P* would result in higher affinity to LDs; however, the opposite was found, where **30** displayed a lower affinity for LDs and a higher affinity for the ER. In addition, the charged probes exhibited mitochondrial localisation and higher cytotoxicity compared to the neutral counterparts, indicating a trend that utilising charged species for enhanced membrane permeability may be at the expense of cytotoxicity and alteration of specificity.

**Fig. 13:**
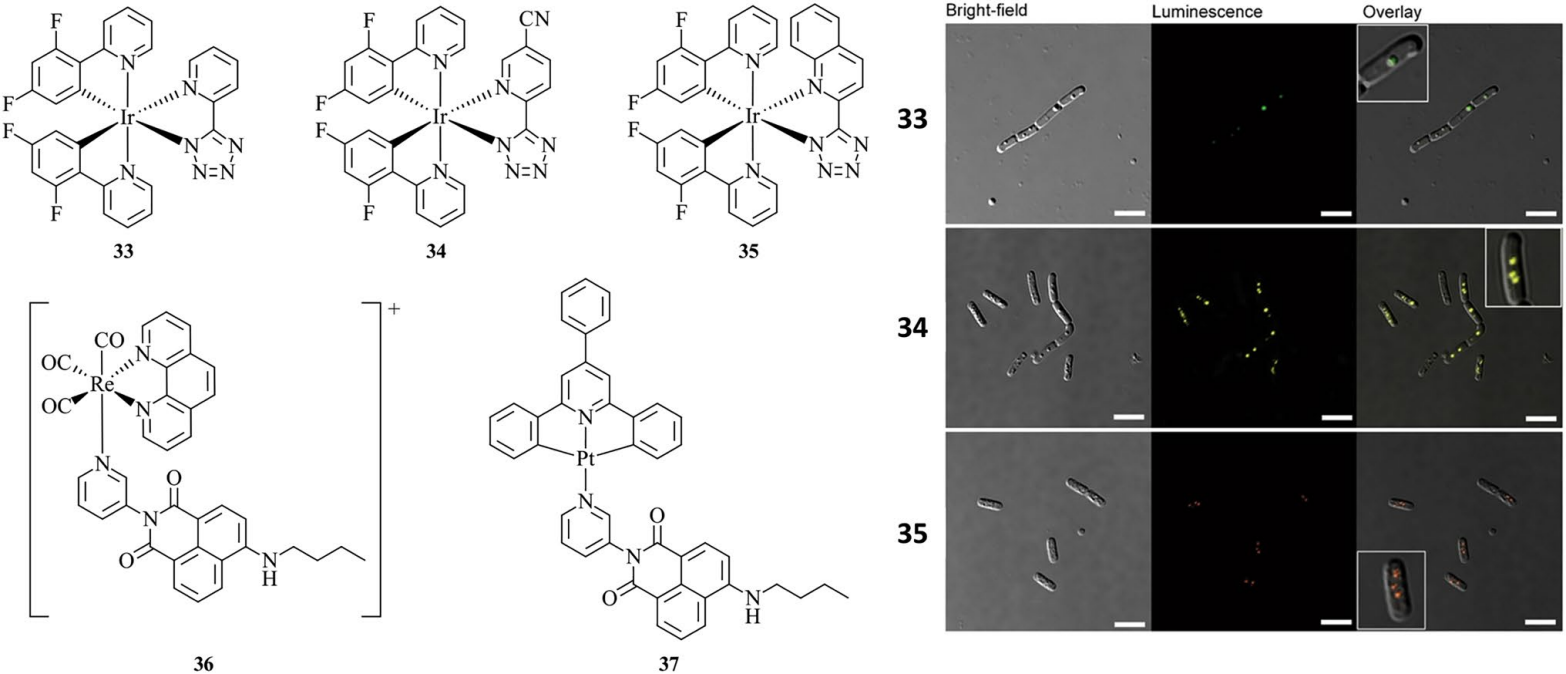
Chemical structures of **33**–**37**. Application of compounds **33**–**35** (yellow) to image lipophilic structures in living bacteria (*B. cereus*) using confocal fluorescence microscopy [66]. Scale bar = 5 µm. Reproduced with permission from Ranieri et al*.* [66]

In addition to targeting lipids in mammalian cells, organometallic probes for targeting lipids in bacteria based on Re(I), Ir(III) and Pt(II) have been explored (Fig. 13). Neutral Ir(III) tetrazolato complexes (**33**–**35**) with tunable emission spanning from 520 to 600 nm with the same excitation wavelength have been shown to efficiently label lipid vacuoles in live *B. cereus* [66]. Subcellular localisation of the nontoxic probes was confirmed with confocal Raman imaging in addition to co-staining studies using BODIPY 493/503. Probes based on Re(I) (**36**) and Pt(II) (**37**) containing a 4-amino-1,8-naphthalimide moiety have been designed and synthesised as potential multi-functional probes for bacteria imaging [71]. However, the low stability of **36** rendered it unsuitable for imaging. The suitability of **37** for structured illumination microscopy (SIM) on live *Bacillus cereus* was demonstrated, revealing LD localisation. The presence of Pt(II) in the probe allowed for subsequent correlative analysis of the sample by nano-scale secondary ion mass spectrometry (nanoSIMS).

**Fig. 14:**
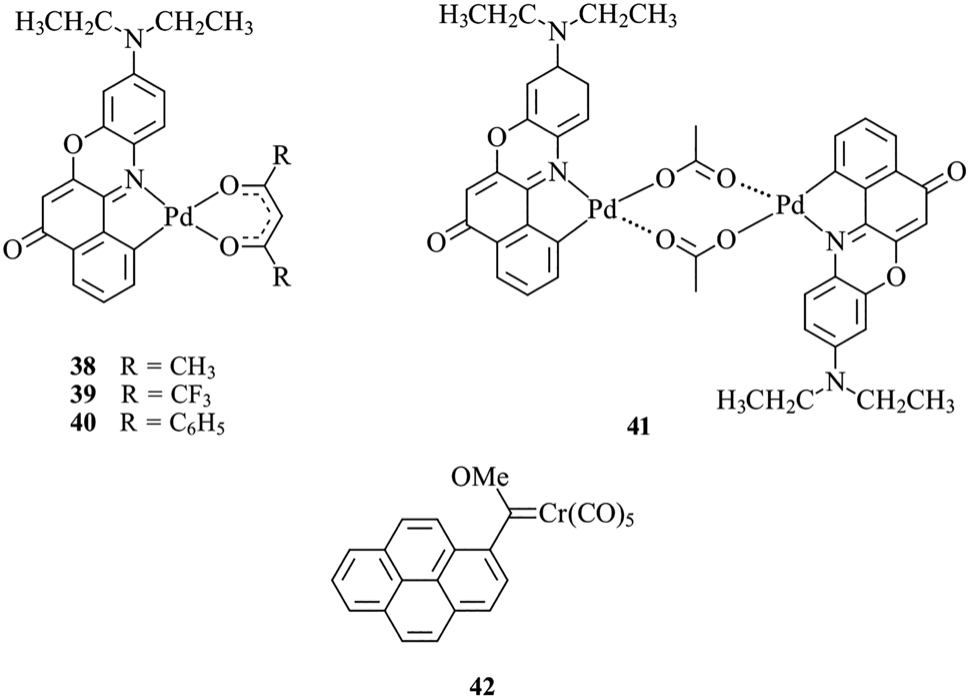
Chemical structures of **38**–**42**

Probes which display solvatochromism change their emission maxima in response to the polarity of their environment. This feature enables monitoring of the lipid polarity surrounding the probe. Nile Red cyclometalated with disubstituted acetylacetonato square planar Pd(II) complexes, such as **38–40**, displays efficient red emission and similar solvatochromic behaviour to Nile Red, indicating a potential ability to distinguish between polar and non-polar lipids (Fig. 14) [121]. The analogous dinuclear complex **41** showed two-photon excitation and was used as CO sensor in vitro and in vivo; however, no investigation on the interaction of this probe with lipids has been reported [122, 123]. A carbon monoxide-releasing Fischer carbene chromium complex (**42**) has been applied to HeLa cells, and co-localisation was observed with both Mitotracker Red and Nile Red [124]. This data suggests the probe localises non-specifically in both the mitochondria and LDs.

##### Probes Incorporating First-Row Transition Metals (Mn)

**Fig. 15:**
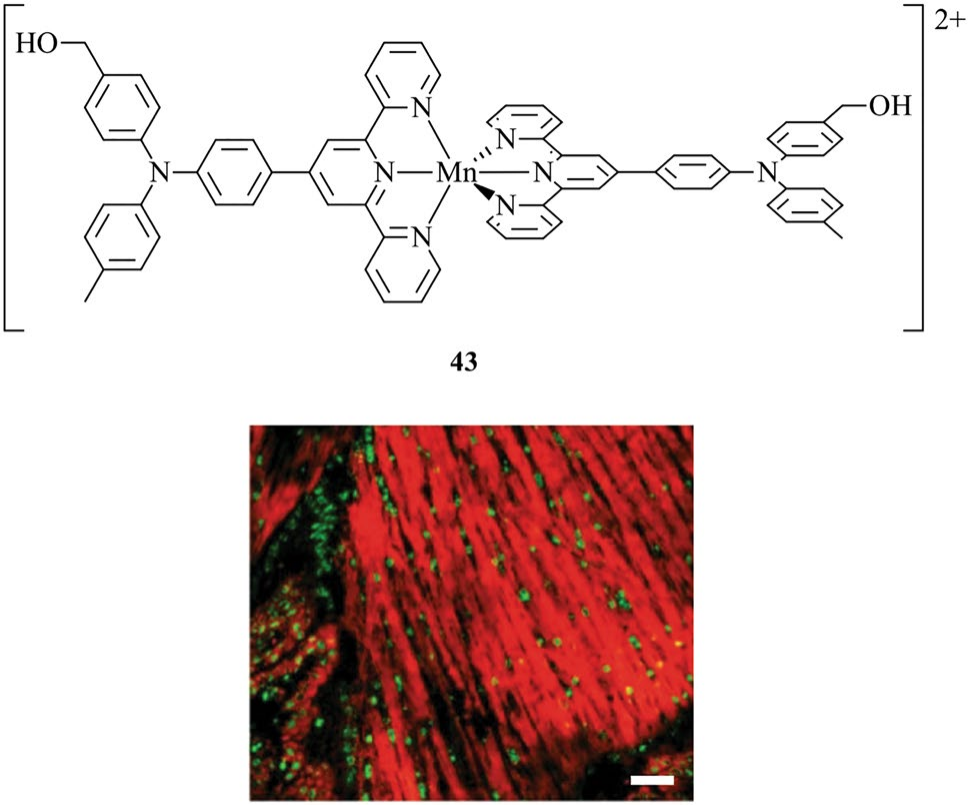
Chemical structure of **43**. Mouse brain incubated with 43 (red) and sectioned into 20 μm slices, co-stained with NucRed (green) and imaged using confocal microscopy. Scale bar = 20 μm. Reproduced with permission from Tian et al. [65]

Tian et al. report a manganese(II) terpyridine metal complex, **43**, which is suitable for both two-photon fluorescence microscopy and MRI (Fig. 15) [65]. Furthermore, it is demonstrated that **43** is suited for stimulated emission depletion (STED). This presents a powerful and unique combination of imaging modalities where super-resolution microscopy reveals structural details at the cellular level, and MRI offers deeper tissue penetration on a larger scale. Complex **43** demonstrates high membrane permeability and low cytotoxicity. Live HepG2 cells were incubated with **43** and co-stained with ER-Tracker Green, showing high co-localisation. When applied to brain tissue, **43** displays strong affinity for parenchymal cells and the myelin sheath within the hippocampus. Myelin specificity was confirmed by co-staining with antibody microtubule-associated protein (MAP2) which label neuronal axons and imaging using STED.

## Conclusion

The microscopy tools available to study lipids in biological systems are rapidly expanding, allowing lipid biology to be explored in unprecedented detail. A critical component of the toolbox to study lipids is the diverse range of lipophilic stains and luminescent probes to reveal cellular and subcellular distributions of lipids. Careful exploitation of the diverse chemistry associated with metal ions, selective tuning of photophysical properties, and analyte specificity has now been achieved to study lipid distribution in cells by numerous research groups. In particular, the use of metal ions provides opportunities to utilise phosphorescence, with associated large Stokes shifts, minimising interference of endogenous autofluorescence encountered in biological samples with time-gated imaging techniques. The high photostability of metal probes which can be exploited for super resolution techniques is beginning to be recognised. While the choice of metal ion is critical to the ability to tune photophysical properties, target specificity is often controlled through selection of ligands. Generic rules for targeting lipids using metal complexes are established, but the detailed relationships between the physicochemical properties and structure of the complex (e.g. charge, lipophilicity and functionalisation) with lipid specificity still requires extensive investigation. What we identify lacking in this area is a set of standard protocols to assess metal-based complexes as lipid probes, or more generally as molecular probes. Experimentations are carried out in different cell lines and conditions, making it very difficult to make comparisons and draw nearer to detailed structure–activity relationships. Furthermore, most newly synthesised compounds and their associated protocols for imaging are optimised for cells, and they do not necessarily translate to the staining of tissue. Given the importance and usefulness of tissue staining, it would be ideal if the set of established standard protocols for the assessment of new molecular probes includes both cells and tissue substrates.

## Abbreviations

cryo-TEM: Cryogenic transmission electron microscopy
DAPI: 4′,6-Diamidino-2-phenylindole
DSPC: 1,2-Distearoyl-sn-glycero-3-phosphocholine
ER: Endoplasmic reticulum
FAD: Flavin adenine dinucleotide
FLIM: Fluorescence lifetime imaging microscopy
FTIR: Fourier transform infrared
ICP-MS: Inductively coupled plasma mass spectrometry
LC: Ligand-centred
LD: Lipid droplet
LF: Ligand field
MLCT: Metal-to-ligand charge transfer
MRI: Magnetic resonance imaging
NAD: Nicotinamide adenine dinucleotide
nanoSIMS: Nanoscale secondary ion mass spectrometry
PLIM: Phosphorescence lifetime imaging microscopy
POSS: Polyhedral oligomeric silsesquioxane
SAXS: Small-angle X-ray scattering
STED: Stimulated emission depletion
